# Maternal and Neonatal Evaluation of Derived Reactive Oxygen Metabolites and Biological Antioxidant Potential in Donkey Mares and Foals

**DOI:** 10.3390/ani11102885

**Published:** 2021-10-03

**Authors:** Micaela Sgorbini, Francesca Bonelli, Giulia Percacini, Anna Pasquini, Alessandra Rota

**Affiliations:** 1 Department of Veterinary Sciences, Veterinary Teaching Hospital, Via Livornese, San Piero a Grado, 56122 Pisa, Italy; francesca.bonelli@unipi.it (F.B.); anna.pasquini@unipi.it (A.P.); alessandra.rota@unipi.it (A.R.); 2 Veterinary Practitioner, 16121 Genova, Italy; g.percacini@libero.it

**Keywords:** oxidative status, BAP, d-ROMs, donkey foal, donkey mare

## Abstract

**Simple Summary:**

The aim of the study was to measure the concentrations of derived reactive oxygen metabolite (d-ROMs) and the biological antioxidant potential (BAP) of donkey mares and foals in order to evaluate the maternal and neonatal oxidative status at delivery and to verify the protective role of the placenta against fetal oxidative stress. A total of 15 Amiata jennies and 17 foals (four foals were born from twin foaling) were included. Immediately after delivery, maternal and foal venous blood samples were drawn from the jugular vein and from one of the two umbilical arteries. Plasma samples were evaluated for d-ROMs and BAP. Lactate was also assessed from whole blood. Statistical analysis was performed. Blood lactate was higher in foals than in their dams. Our results support the hypothesis that the placenta may be a protective barrier against oxidative damage and that the antioxidant system is not well formed at birth in donkey foals.

**Abstract:**

Our aim was to measure the concentrations of derived reactive oxygen metabolite (d-ROMs) and biological antioxidant potential (BAP) of donkey mares and foals at delivery and to verify the protective role of the placenta against fetal oxidative stress. A total of 15 Amiata jennies with a physiological gestation length and delivery were included together with 17 foals (two twin foalings). After delivery, maternal and foal venous blood samples were collected along with blood from the artery. Circulating lactate and plasma d-ROMs and BAP were evaluated. The Wilcoxon test for paired data was applied to verify differences in d-ROMs and BAP values, while the Spearman test was used to evaluate correlations. A significantly higher d-ROMs concentration was found in jennies compared to their foals, and to the umbilical artery blood. The BAP was higher in jennies than in their foals, but no differences were observed in the umbilical artery blood. No difference was found between foals and their umbilical cord. Blood lactate was higher in foals than in their dams. Positive correlations were found between mares and umbilical cord for BAP and d-ROMs, and between mares and foals and umbilical cord for BAP. In conclusion, the placenta may be a protective factor for the fetus. As with equine foals, the antioxidant system of donkey foals does not seems to be effective at birth.

## 1. Introduction

Reactive oxygen species (ROS) are the different forms of activated oxygen that play both positive and negative roles in biological mechanisms [1]. Controlled ROS production is indispensable to cellular signal messengers as it regulates gene expression and downstream cellular activities [2]. In fact, it regulates several signal transduction pathways: the activity of enzymes is involved in signaling cell growth and differentiation, and mediating inflammation by stimulating cytokine production and eliminating pathogens and foreign particles [3]. Oxidative stress (OS) occurs when ROS generation is not adequately balanced by antioxidant systems. High ROS concentrations can cause irreversible oxidative damage in biomolecules, and an increased number of ROS disrupt the redox homeostasis, causing oxidative stress [4,5,6,7]. The antioxidant defense, consisting of enzymes, vitamins and oligo-elements, reduces ROS-related damage [8].

In human literature, pregnancy is characterized by high energy requirements and an increase in oxygen consumption by both the placenta and fetus [2,8]. Thus, the placenta is a site of oxygen metabolism that continuously produces OS. In order to control the overproduction of ROS, the placenta produces abundant antioxidants [2]. 

The increased metabolism during pregnancy induces an overproduction of ROS [9,10]. Moreover, some prenatal conditions that enhance oxidative stress linked to inflammation or hypoxia have been associated with impaired fetal growth and preterm delivery in up to 50% of cases in humans. These pregnancy-related disorders are caused by the imbalance between the protective (antioxidant system) and destructive mechanisms (ROS activity) [11]. 

OS is strongly involved in the pathogenesis of many newborn diseases, and higher concentrations of OS markers have been found in the umbilical cord blood in babies affected, for example, by necrotizing enterocolitis or hypoxic-ischemic encephalopathy. This is due to the low efficiency of neonatal antioxidant systems, which are unable to counteract the effect of ROS. Higher concentrations of OS markers have also been found in the amniotic fluid in pregnancies complicated by preeclampsia, fetal growth restriction, maternal obesity, and gestational diabetes. The enhanced OS is linked to placental inflammation or hypoxia [7,11]. 

Despite newborn babies also being susceptible to oxidative stress, several studies have shown the important role played by the placenta in protecting the fetus against O_2_ toxicity during pregnancy and delivery [6,12]. In humans, placental ischemia/hypoxia can disrupt the balance between ROS and antioxidants, leading to damage in proteins, lipids, and DNA in the fetus [6,13]. 

In veterinary medicine, antioxidative or oxidative status has been studied during pregnancy, lactation, and at delivery in cows [14,15,16,17,18,19,20,21] and small ruminants [22,23,24]. In ruminants, the oxidative status can be used as a marker for evaluating and preventing metabolic disorders characterized by z negative energy balance during pregnancy and peri-partum period [14,16,17,18,21]. These findings are crucial, especially if we consider that the OS during pregnancy and delivery can cause not only diseases (i.e., gravidic toxemia, fetal growth retardation, placental retention, and mastitis) but also a reduction in milk production or post-partum metabolic diseases. 

In newborn calves, the oxidant and antioxidant systems were evaluated in a cohort of 14 calves at birth and four times after birth up to three weeks of age. The authors found that the concentration of free radicals in newborn calves was 30% higher than in their mothers, suggesting an increase in ROS after birth. The ROS concentration decreases thereafter during the first week of life, probably due to the induction of the antioxidant defense in newborn calves. In fact, the authors found a higher ferric reducing ability of plasma (FRAP) concentration in calves compared to their mothers. The antioxidant enzyme activity found in newborn calves thus seems to be well prepared to deal with OS at birth [16].

The oxidative status has also been studied in horses [25,26,27,28,29,30,31,32,33], and there are also a few studies on donkeys [34,35,36]. In horses, studies assessing oxidative status in relation to exercise reported higher concentrations of derived reactive oxygen metabolites (d-ROMs) after sub-maximal exercise on a treadmill, compared to rest [26,27]. d-ROMs and biological antioxidant potential (BAP) also increased in an endurance test when horses underwent an intense exercise, thus indicating an imbalance between oxidants and antioxidants mainly characterized by an increased antioxidant response [28]. OS markers have also been evaluated in horses after surgery in which markers significantly increased after castration, suggesting a decrease in the antioxidant potential and causing OS [29]. During pregnancy, mares supplemented with a high amount of alfa-tocopherol did not show oxidative stress during the peripartum period, suggesting an increase in the antioxidant potential [30]. In mares and their foals, d-ROMs and BAP were evaluated at birth to assess perinatal oxidative status and to compare the results with the human and veterinary literature [25]. Oxidative markers were evaluated in healthy foals and foals affected by *R. equi* pneumonia. The authors found an increase in d-ROMs concentration both in the blood and exhaled breath condensate of sick foals compared to the healthy group [31].

In donkeys, the literature on the evaluation of the oxidative status is scarce. Oxidative stress has been evaluated in *Strongylus* spp.-infected donkeys, and the authors found higher concentrations of OS biomarkers before treatment [35]. Dietary supplementation with a natural polyphenol (verbascoside) has been reported to decrease ROM concentration and increase vitamin E levels in jennies and their suckling foals [36]. 

To the best of our knowledge, the oxidative status in healthy donkey mares and their foals has not been evaluated. The aim of the present study was thus to measure the concentrations of d-ROMs and BAP in donkey mares and foals in order to evaluate the maternal and neonatal oxidative status at delivery and verify the protective role of the placenta against fetal oxidative stress.

## 2. Materials and Methods

A total of 15 Amiata jennies aged 3–16 years (median age 9 years old) old and 17 neonatal foals (4 foals were from twin foalings) were included in the present study. The animals were housed on a stud farm where all the deliveries took place. Ethical approval to conduct the study was obtained from the Ethics Committee on Animal Experimentation of the University of Pisa (prot. N. 2825/14), and owner consent was obtained. 

Jennies were included according to the following criteria: (1) pregnancy length longer than 353 days [37]; (2) eutocic, unassisted delivery. Attendance at birth was ensured by daily monitoring of mammary development and, when mammary secretion was present, by measuring the milk calcium concentration every 24 h at 6:00 p.m. using a commercially available kit (FoalWatch^®^ Titres for Daytime Foaling Management Chemetrics, Inc., Calverton, VA, USA). Jennies were constantly supervised 24 h/day when calcium in the colostrum was over 200 ppm [38]. Foals were evaluated for the APGAR score [39].

Immediately after delivery, maternal and foal venous blood samples were drawn from the jugular vein and collected by one operator into lithium-heparinized test tubes (FL Medical, Padua, Italy). Simultaneously, a heparinized blood sample was drawn from one of the two umbilical arteries by a second operator. Heparinized samples were centrifuged at 3000× *g* for 10 min within 20 min of sampling, as recommended by the manufacturer. Plasma was frozen at −20 °C and analyzed in a single batch. 

All plasma samples were evaluated for d-ROMs (d-ROMs test, Diacron srl, Grosseto, Italy) and BAP (BAP test, Diacron srl, Grosseto, Italy). The analysis was carried out using a spectrophotometer (Slim, SEAC, Florence, Italy), following the manufacturer’s instructions. The d-ROMs test measures the blood concentration of hydroperoxides, a class of chemical oxidant species belonging to the group of ROM. In brief, in the d-ROMs test, reactive oxygen metabolites, in the presence of iron, are able to generate alkoxyl and peroxyl radicals, according to the Fenton reaction. Such radicals can then oxidize an amine (N,N-dietylparaphenylendiamine), thus producing a pink-colored derivative, which is photometrically quantified at 505 nm. The d-ROMs concentration is directly proportional to the color intensity and is expressed as Carratelli Units (1 CARR U = 0.08 mg hydrogen peroxide/dL). The BAP is a photometric test that measures the plasma biological antioxidant potential as the capacity of the plasma sample to reduce iron from ferric (Fe^3+^) to ferrous form (Fe^2+^). In the BAP test, the addition of a plasma sample to a colored solution, obtained by mixing a ferric chloride solution with a thiocyanate derivative solution, causes a discoloration, whose intensity is measured photometrically at 505 nm and is proportional to the ability of the plasma to reduce ferric ions. The results are expressed as mmol/L of reduced ferric ions. The stability of the d-ROMs and BAP tests in stored samples was evaluated in previous studies [40,41]. Lactate was also assessed in whole blood using a dedicated point-of-care testing device (Accutrend Lactate^®^, Micralab srl, MI, USA) and expressed as mmol/L.

### Statistical Analysis

Data were analyzed for distribution by the Shapiro–Wilk test. Since not all the data showed a Gaussian distribution, we performed the statistical analysis using non-parametric tests and expressed the results as median, minimum, and maximum values. The Wilcoxon matched-pairs signed rank test for paired data was applied to verify differences in d-ROMs and BAP values between jennies, their foals, and the umbilical cord. The Mann–Whitney test was applied to assess differences in lactate concentrations between jennies and foals.

The Spearman test with a two-tailed p-value and a confidence interval of 95% was performed to evaluate correlation between mares, umbilical cord, and foals for d-ROMs and BAP values, and between d-ROMs and blood lactate, both for mares and foals. Results obtained from twins were reported but not included in the statistical analysis. Results were considered to be statistically significant for *p* < 0.05 (GraphPad Prism, 8.0, San Diego, CA 92108, USA).

## 3. Results

Of the 17 donkey foals, 12/17 were fillies (of which one was a second-born twin) and 5/17 were colts (of which one was a second-born twin). The APGAR score in non-twin foals ranged between 7 and 8/8, while the range was 6–7/8 in twins. The APGAR was 6/8 in both twins born first and 7/8 in both twins born second. 

Table 1 reports the results on d-ROMs and BAP concentrations in jennies, umbilical artery blood, and non-twin foals, and blood lactate in jennies and non-twin foals. Table 2 reports the results obtained in the two pairs of twins.

Statistical analysis showed a significantly higher d-ROMs concentration in jennies compared to their foals (*p* = 0.0105), and to the umbilical artery blood (*p* = 0.0002); however, there was no difference between foals and their umbilical cord (*p* = 0.1677). BAP concentration was higher in jennies compared to their foals (*p* = 0.0129) and to umbilical artery blood (*p* = 0.0203), but no differences were found between foals and umbilical cord (*p* = 0.2163). Blood lactate was higher (*p* = 0.0002) in foals with respect to their dams. 

Positive correlations were found between mares and umbilical cord (r = 0.569, *p* = 0.045) for d-ROMs concentrations and between mares and foals (r = 0.544, *p* = 0.014) and umbilical cord (r = 0.758, *p* = 0.004) for BAP concentration. No correlation was found between d-ROMs and blood lactate in the jennies or the foals.

## 4. Discussion

Oxidative stress occurs when there is an imbalance between antioxidant- and oxidant-generating systems, resulting in cellular injury or the activation of pathological pathways. It can develop under para-physiological conditions, such as physical exercise. In fact, oxidative stress has been widely investigated in sport horses [42]. However, there have only been few reports regarding OS in mares during pregnancy and delivery [25,32], and, to the best of our knowledge, there are no published data for donkeys. 

We thus investigated antioxidant- and oxidant-generating systems in a group of jennies and their foals by measuring the d-ROMs and BAP concentrations. In women, the concentrations of oxidative agents are higher in maternal blood compared to cord blood at birth [9,43]. Moreover, the concentration of lipoperoxides increases as pregnancy advances and peaks at delivery. Human pregnancy is associated with oxidative stress due to the increased demand for oxygen, but newborns are protected by an increase in the total antioxidant system (TAS) of their mothers [9]. 

In our previous study on horses [25], the d-ROMs values were higher in maternal blood than in umbilical artery blood and foals’ blood at birth, in line with humans. For donkeys, we found higher concentrations of d-ROMs in jennies compared to both umbilical cord and their foals, confirming a similar trend between donkeys and humans. In women, placenta seems to secrete oxidative factors into the maternal circulation and not into the fetal circulation [44,45]. Our results support the hypothesis that the placenta could prevent excessive OS by producing higher oxidative agents in the mother compared to the fetus [44,45], as also found in horses [25].

In the present study, we found higher BAP concentrations in donkey mares than in their foals, in line with our study on horses [25]. In fact, a higher BAP concentration was found in mares than in either their umbilical artery blood or their foals [25]. In our previous study, we hypothesized that the equine foals’ antioxidant system was not completely effective at birth due to the poor reserve of endogenous antioxidants and the inability to upregulate the defense mechanisms in response to the increase in ROS production, as reported in newborn humans [7,9]. The low efficiency of the antioxidant system in foals could lead to oxidative damage, as reported for newborn humans. In fact, newborns, especially preterm infants, are at high risk of OS at birth, and they are very susceptible to oxidative damage by ROS because the extrauterine environment is richer in oxygen than the intrauterine one [7,11]. 

In the present study, we found differences between jennies and their foals and the umbilical artery blood. Our results for donkeys agree with what we found previously in horses. Our findings are thus likely due the immaturity of the donkey foals’ antioxidant system at birth. Our results for horses and donkeys are in contrast to findings reported in newborn calves, in which the antioxidant enzyme activity seems to be well prepared to handle the OS at birth [16].

Compared to horses [25], the d-ROMs and BAP values obtained in donkeys using the same methods were higher, both for mothers, umbilical cords, and foals. These differences could be related to the different species studied. This hypothesis needs confirmation, as these, to our knowledge, are the first data of d-ROMs and BAP concentrations in donkeys. 

Lactate concentrations in donkey foals and mares are slightly higher than in horses, as in equine species [46].

A correlation between mares and umbilical cord was found for the d-ROMs values. These results are in line with findings in humans that suggested that a high oxidative status in the mother corresponded to a high oxidative state of the umbilical cord blood and newborn [47]. A correlation was also found between jennies, foals, and umbilical cord, for BAP values. The role and the production pathways of antioxidative activity in newborns have still not been clarified. Some studies performed on humans support the existence of antioxidant activity in fetal circulation [48,49], suggesting that neonates just after birth have already been endowed with the ability to cope with OS [50]. Another hypothesis could be that these findings are related to the transport, although limited, of antioxidants from the mother to their foals through the placenta [51].

No correlation was found between the concentrations of d-ROMs and blood lactate either in jennies or foals. This result partially agrees with what we previously found for horses [25], where a positive correlation was found between blood lactate and d-ROMs in mares but not in foals. Lactate induces the production of mitochondrial ROS [52]. In mares, the increase in lactate for the high energy demand at delivery was followed by a higher concentration of d-ROMs [25], but this did not appear to happen in jennies.

In the present study, the Apgar score in twins was higher in both the second-born foals with respect to the first-born ones: both in the twins born first (Apgar value 6/8) and in the twins born second (Apgar value 7/8). In humans, multiple pregnancies are associated with an increased production of reactive oxygen species and decreased activity of antioxidant defenses, and the development of the OS [53,54,55]. Moreover, in humans a reduction in the antioxidant and an increment in oxidative activities were found in the second born twin compared to the first born one [53].

In our study, due to the low number of twins enrolled, we were unable to compare the oxidant and antioxidant parameters between twins and others or between the first and second born twin. However, the d-ROMs, BAP, and lactate values in the twins seem to be similar to what we found in the non-twin donkey foals, and all twins showed good to optimal viability (Apgar value 6–7/8). The delivery of twins does not seem to be a cause of oxidative imbalance, and the viability does not seem to be impaired in the second born foals.

Further studies are needed to verify possible differences in twins compared to single born donkey foals. However, it is relatively more common for jennies to deliver healthy twins than for mares [56]. This may be explained by a more efficient placenta since, compared with mares, jenny chorioallantois has a higher concentration of microcotyledons and an extensive branching of the villi [57].

## 5. Conclusions

In conclusion, the placenta may protect the fetus against OS at delivery. However, as in horses, the antioxidant system does not seem to be effective in donkey foals. We plan further studies to investigate the implications of these findings in donkey foals affected by neonatal diseases.

## Figures and Tables

**Table 1:** d-ROMs and BAP concentrations in dams, umbilical cord, and foals, and blood lactate in foals and their dams expressed as median, minimum, and maximum values.

Parameters	Jennies	Umbilical Cord	Non-Twin Foals
D-ROMs (U.Carr.)	271.6 ^a^ 188.7–311.1	157.5 ^b^ 113–201	184.5 ^b^ 111–389.6
BAP (μmol/L)	2752 ^a^ 1284–3068	2655 ^b^ 1038–2942	2461 ^b^ 1244–2740
Blood lactate (mmol/L)	3.5 ^a^ 2.2–4.8	-	5.2 ^b^ 4.3–6.9

BAP, biological antioxidant potential; d-ROMs, derived Reactive Oxygen Metabolites. ^a,b^: The different lower-case letters denote a significant difference (a ≠ b: *p* < 0.05).

**Table 2:** d-ROMs and BAP concentrations in the dams, umbilical cords, and twin-foals, and blood lactate in twin-foals and their dams.

		**D-ROMs (U.Carr.)**			
		**UC-F1**	**UC-F2**	**F1**	**F2**
**J1**	268.2	169.5	169.5	157.3	228.3
**J2**	209.3	160.9	160.9	251.2	250
		**BAP (μmol/L)**			
		**UC-F1**	**UC-F2**	**F1**	**F2**
**J1**	2743	2908	2974	2677	2809
**J2**	2611	2316	2835	2619	2569
		**Blood lactate (mmol/L)**			
		**UC-F1**	**UC-F2**	**F1**	**F2**
**J1**	3.3	-	-	5.1	5.8
**J2**	3.2	-	-	5.6	6.8

J1: jenny 1; J2: jenny 2; UC-F1: umbilical cord foal first born; UC-F2: umbilical cord second born; F1: first born; and F2: second born.

## Data Availability

The data presented in this study are available on request from the corresponding author.
